# Selection of Orthologous Genes for Construction of a Highly Resolved Phylogenetic Tree and Clarification of the Phylogeny of Trichosporonales Species

**DOI:** 10.1371/journal.pone.0131217

**Published:** 2015-08-04

**Authors:** Masako Takashima, Ri-ichiroh Manabe, Wataru Iwasaki, Akira Ohyama, Moriya Ohkuma, Takashi Sugita

**Affiliations:** 1 Japan Collection of Microorganisms, RIKEN BioResource Center, Koyadai, Tsukuba, Ibaraki, 305–0074, Japan; 2 Division of Genomic Technologies, RIKEN Center for Life Science Technologies, Suehiro-cho, Tsurumiku, Yokohama, Kanagawa, 230–0045, Japan; 3 Department of Biological Sciences, Graduate School of Science, the University of Tokyo, Yayoi, Bunkyoku, Tokyo, 113–0032, Japan; 4 Planning, in silico biology, inc., SOHO Station 706, 24–8 Yamashita-cho, Naka-ku, Yokohama, Kanagawa, 231–0023 Japan; 5 Department of Microbiology, Meiji Pharmaceutical University, Kiyose, Tokyo, 204–8588, Japan; Leibniz Institute DSMZ-German Collection of Microorganisms and Cell Cultures, GERMANY

## Abstract

The order Trichosporonales (Tremellomycotina, Basidiomycota) includes various species that have clinical, agricultural and biotechnological value. Thus, understanding why and how evolutionary diversification occurred within this order is extremely important. This study clarified the phylogenetic relationships among Tricosporonales species. To select genes suitable for phylogenetic analysis, we determined the draft genomes of 17 Trichosporonales species and extracted 30 protein-coding DNA sequences (CDSs) from genomic data. The CDS regions of *Trichosporon asahii *and *T*. *faecale *were identified by referring to mRNA sequence data since the intron positions of the respective genes differed from those of *Cryptococcus neoformans* (outgroup) and are not conserved within this order. A multiple alignment of the respective gene was first constructed using the CDSs of *T*. *asahii*, *T*. *faecale* and *C*. *neoformans*, and those of other species were added and aligned based on codons. The phylogenetic trees were constructed based on each gene and a concatenated alignment. Resolution of the maximum-likelihood trees estimated from the concatenated dataset based on both nucleotide (72,531) and amino acid (24,173) sequences were greater than in previous reports. In addition, we found that several genes, such as phosphatidylinositol 3-kinase *TOR1 *and glutamate synthase (NADH), had good resolution in this group (even when used alone). Our study proposes a set of genes suitable for constructing a phylogenetic tree with high resolution to examine evolutionary diversification in Trichosporonales. These can also be used for epidemiological and biogeographical studies, and may also serve as the basis for a comprehensive reclassification of pleomorphic fungi.

## Introduction

The order Trichosporonales was proposed by Fell et al. [1] and, in “The Yeasts, A Taxonomic Study” 5^th^ ed., it includes the genera *Trichosporon* (37 species) [2] and *Cryptotrichosporon* (1 species) [3], as well as some species of highly polyphyletic genera, *Bullera* (3 of 41 species listed) [4] and *Cryptococcus* (10 of 70 species listed) [5]. Since the type species of the genera *Bullera* and *Cryptococcus* were assigned to the Tremellales, species within these two genera in the Trichosporonales are misleading taxonomically.

**Fig 1:**
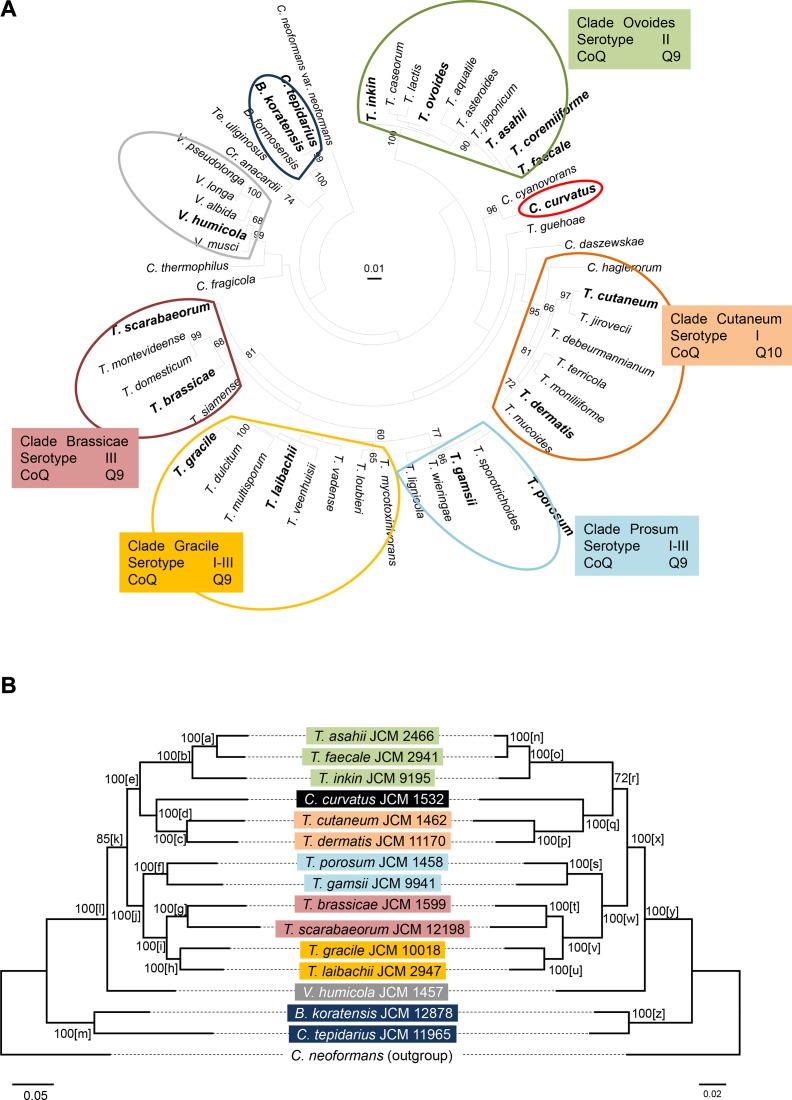
Phylogenetic trees of *Trichosporon* species and related taxa based on (a) D1/D2 LSU rRNA gene sequences, (b) nucleotide sequences of 30 concatenated genes, and (c) amino acid sequences of 30 concatenated genes. The tree in (a) was constructed using the maximum likelihood method based on the Tamura-Nei model [44]. The names of clades are based on those in Fig. 161.1 in *The Yeasts*, *A Taxonomic Study*, 5^th^ ed. [2]. Boldface indicates that the species was used for the 30-gene concatenation analysis. The trees in (b) and (c) were constructed using the maximum likelihood method based on the Tamura-Nei model [44] for (b) and a JTT matrix-based model [45] for (c). Numerals on each node represent the percentages from 100 replicating bootstrap samplings (frequencies of less than 60% are not shown) [46]. Letters in brackets after the bootstrap values in (b) and (c) indicate the node name (see Table 3). Positions used: (a) 604 nucleotides, (b) 72,531 nucleotides, (c) 24,173 amino acids. Highest log likelihood: (a) -3894.3736, (b) -611997.4313, (c) -216445.1306. *B*., *Bullera*; *C*., *Cryptococcus*; *Cr*., *Cryptotrichosporon*; *T*., *Trichosporon*; *Te*., *Tetragoniomyces*; *V*., *Vanrija*.

The genus *Trichosporon*, as it is defined at this time, is polyphyletic and divided into five clades; namely, Brassicae, Cutaneum, Gracile, Ovoides and Porosum. Each is traditionally characterized based on a combination of serological properties (serogroup) and the major ubiquinone (as the phenotype), as shown in Fig 1a. In the *Cryptococcus* species mentioned above, Weiss et al. [6] recently reported the amended concept of the genus *Vanrija* [7] by following several discussions on this group [2, 3, 5], and proposed the use of *Vanrija* as the genus name for *Cryptococcus humicola* and phylogenetically related species.

Clinically, biotechnologically and agriculturally important species are included in this order: approximately one-third of species in the genus *Trichosporon* are associated with infections or allergies in humans [2]; *Trichosporon asahii* (Ovoides clade) is the causative agent of trichosporonosis and summer-type hypersensitivity pneumonitis (SHP) [8]. *Trichosporon mycotoxinivorans* (Gracile clade) detoxifies mycotoxins, such as ochratoxin A and zearalenone [9], and *Trichosporon porosum* (Porosum clade) is thought to function in the global carbon cycle because it possesses *CbhI* genes for cellobiohydrolase [10] and oleaginous activity [11]. Additional oleaginous species have been reported among the Trichosporonales, including *Cryptococcus curvatus* [12], *Vanrija albida* [13], *Vanrija musci* [13], *Trichosporon cacaoliposimilis* (Gracile clade) [14] and *Trichosporon oleaginosus* (Cutaneum clade) [14]. Some species show cellulase activity, and most species can assimilate various compounds including aromatic and aliphatic compounds [2]. Since the expression (as shown above) and implied potential activities have been characterized in this order, understanding why and how this evolutionary diversification occurred is important. We have performed various studies on the genus *Trichosporon* and related species, including intra- and inter-species diversity; however, the phylogenetic relationships and evolutionary processes in this order remain unclear [2].

This study was performed to clarify the phylogenetic relationships among Trichosporonales species, especially basal species and clusters within this lineage. The draft genomes of 17 Trichosporonales species were determined, 30 orthologous protein-coding DNA sequences (CDSs) were selected, and phylogenetic trees were constructed based on the respective genes and a concatenated alignment. When selecting CDSs suitable for this analysis, the draft transcriptomes of *T*. *asahii* and *T*. *faecale* were used to identify coding regions for these species. Each CDS of *T*. *asahii* and *T*. *faecale* was aligned with that of *Cryptococcus neoformans* (outgroup), and those of other species were added and aligned based on codons, because the intron position of the respective genes is not conserved in this order.

## Results and Discussion

### Gene selection

A summary of the draft genome sequences is shown in Table 1. The obtained genomic size of *T*. *asahii* was similar to other recently published data (ALBS01000000 and AMBO01000000). The range of genomic sizes of Trichosporonales species, estimated based on the total length of contigs in this study, ranged from 18.2 Mb (*C*. *curvatus*) to 42.2 Mb (*Trichosporon coremiiforme*). We did not observe a trend between genome size and phylogenetic position or morphology in this study.

**Table 1:** Strains used in this study.

Scientific name	Strain no.	Source*^1^	# Contigs	Total length(bases)	N50(bases)	Maximum(bases)	Average (bases)
*Trichosporon asahii*	JCM 2466	nail of a psoriasis patient, Fukuoka, Japan	1,262	24,495,558	77,499	335,598	19,410
*Trichosporon brassicae*	JCM 1599	cabbage, T. Nakase, Tokyo, Japan	988	23,545,754	46,311	203,527	23,832
*Trichosporon coremiiforme*	JCM 2938	lesion on the head of a farmer, Costa Rica	3,232	42,172,195	26,704	150,431	13,048
*Trichosporon cutaneum*	JCM 1462	skin lesion	3,076	22,615,927	14,018	146,985	7,352
*Trichosporon dermatis*	JCM 11170	infected human skin, Tübingen, Germany	1,016	23,295,971	46,612	233,244	22,929
*Trichosporon faecale*	JCM 2941	human feces, A.C. Batista	551	24,482,466	94,928	366,476	44,433
*Trichosporon gamsii*	JCM 9941	moist humus around roots, W. Gams, Sierra Nevada de Santa Marta, Colombia	7,995	25,860,364	17,022	114,205	3,235
*Trichosporon gracile*	JCM 10018	sour milk, Germany	1,672	24,039,830	29,596	175,298	14,378
*Trichosporon inkin*	JCM 9195	human skin with tinea cruris caused by *Trichophyton*, L. do Carmo-Sousa	3,680	19,793,539	17,312	173,610	5,379
*Trichosporon laibachii*	JCM 2947	soil	3,380	30,595,110	21,469	138,973	9,052
*Trichosporon ovoides*	JCM 9940	hair of a white piedra patient, A. Lasagni	3,336	39,800,440	45,378	382,552	11,931
*Trichosporon porosum*	JCM 1458	exudate of English yew (*Taxus baccata*), Hamburg, Germany	1,838	25,943,715	30,685	146,128	14,115
*Trichosporon scarabaeorum*	JCM 12198	gut of a larval scarab beetle, South Africa	2,755	30,549,951	30,687	133,129	11,089
*Bullera koratensis*	JCM 12878	leaf of plant, Thailand	1,443	25,088,877	33,905	163,727	17,387
*Cryptococcus curvatus*	JCM 1532	sputum of a patient with tuberculosis	531	18,156,997	81,043	240,381	34,194
*Cryptococcus tepidarius*	JCM 11965	water in stream, Owakudani, Hakone, Japan	3,328	22,106,340	11,260	65,094	6,643
*Vanrija humicola*	JCM 1457	heath soil in Switzerland	3,111	22,380,125	15,011	154,669	7,194

*^1^ data from [2, 5] and http://jcm.brc.riken.jp/en/catalogue_e.

The candidate genes for phylogenetic analysis selected in this study are shown in Table 2. These 30 genes were selected as described in the Materials and Methods without consideration of function. They are orthologous genes shown in the Microbial Genome Database for Comparative Analysis (MBGD) (http://mbgd.nibb.ac.jp). Notably, we found that the genomes of *T*. *coremiiforme* and *T*. *ovoides* occasionally include two paralogs for some of the studied genes, whereas those of other species contained only one (Table 2). Even including the two paralogs from *T*. *ovoides* and *T*. *coremiiforme*, all phylogenetic trees based on a single gene showed that the former constituted a subclade with *T*. *inkin* and the latter with *T*. *asahii* and *T*. *faecale* (data not shown). This topology was the same as reported previously [2], indicating that the paralogous genes were not transferred from phylogenetically distant species. Therefore, these two species were excluded from the robustness analyses of the tree topology.

**Table 2:** Genes used in this study.

Product	Homolog to: (locus_tag of *C*. *neoformans)*	Function category*^1^	Sequence accession numbers for 17 species	Number of paralogs detected in^†^	
				*T*. *coremiiforme*	*T*. *ovoides*
methylenetetrahydrofolate reductase (NADPH)	CNL04820	Amino acid biosynthesis [1.2]	AB920057-AB920077	2	2
argininosuccinate lyase	CNC04420	Amino acid biosynthesis [1.4]	AB919708-AB919727	2	1
glutamate synthase (NADH)	CNJ02910	Amino acid biosynthesis [1.4]	AB920036-AB920056	2	1
orotidine monophosphate pyrophosphorylase (*URA5*)	CNG03730	Purines, pyrimidines, nucleosides, and nucleotides [2.4]	AB919901-AB919919	2	2
pyruvate carboxylase	CNF00650	Fatty acid, phospholipid and sterol metabolism [3.1], Transport and binding proteins [7.4], Other categories [14]	AB919845-AB919864	1	2
actin	CNA04650	Central intermediary metabolism [5.2]	AB919611-AB919629	2	2
adenylyl-sulfate kinase	CNE03380	Central intermediary metabolism [5.5]	AB919806-AB919824	2	2
translation elongation factor 1-α	CNM01300	Central intermediary metabolism [5.5], Translation [10.4]	AB920098-AB920117	1	2
adenosylhomocysteinase	CND00240	Central intermediary metabolism [5]	AB919748-AB919768	2	2
1,3-beta-glucan synthase	CNN02320	Central intermediary metabolism [5]	AB920158-AB920176	2	2
citrate synthase	CNA00510	Energy metabolism [6.13]	AB919588-AB919610	2	2
vacuolar ATP synthase	CNI01180	Energy metabolism [6.4]	AB919979-AB919997	2	2
phosphoenolpyruvate carboxykinase	CNI03590	Energy metabolism [6.8]	AB919998-AB920016	2	1
malate synthase	CNH02910	Energy metabolism [6]	AB919920-AB919940	2	2
isocitrate lyase	CNH03280	Energy metabolism [6]	AB919941-AB919961	1	2
plasma membrane H(+)-ATPase 1	CNN01260	Transport and binding proteins [7.4]	AB920136-AB920157	2	2
ATP-binding cassette (ABC) transporter	CND00300	Transport and binding proteins [7.6]	AB919769-AB919786	2	1
ATP dependent DNA helicase	CNB05360	DNA replication, restriction, modification, recombination, and repair [8.2]	AB919670-AB919688	2	2
DNA replication licensing factor *cdc19* (cell division control protein 19)	CNG02380	DNA replication, restriction, modification, recombination, and repair [8.2]	AB919883-AB919900	1	2
DNA-dependent RNA polymerase II *RPB140* (*RPB2*)	CND03540	Transcription [9.2]	AB919787-AB919805	1	2
DNA-directed RNA polymerase ii largest subunit, putative (*RPB1*)	CNE03720	Transcription [9.2]	AB919825-AB919844	2	2
DNA-directed RNA polymerase	CNI00420	Transcription [9.2]	AB919962-AB919978	1	1
*RAS2*	CNJ01920	Translation [10.5]	AB920017-AB920035	1	2
glycogen synthase kinase 3	CNB00720	Regulatory functions [11.1]	AB919630-AB919650	2	2
mitogen-activated protein kinase	CNC06590	Regulatory functions [11.1]	AB919728-AB919747	2	2
phosphatidylinositol 3-kinase *TOR1*	CNF03740	Regulatory functions [11.1]	AB919865-AB919882	1	2
chaperone	CNM01520	Cellular processes [13.3]	AB920118-AB920135	1	2
alpha tubulin	CNB02810	Cellular processes and signaling; Cytoskeleton (COG)*^2^	AB919651-AB919669	2	2
beta1-tubulin, putative	CNC03260	Cellular processes and signaling; Cytoskeleton (COG)*^2^	AB919689-AB919707	2	2
eukaryotic translation initiation factor 3 subunit 6	CNL05160	Unknown (KEGG)*^2^	AB920078-AB920097	2	2

*^1^ Data from Microbial Genome Database for Comparative Analysis (MBGD) (http://mbgd.nibb.ac.jp). Numerals in brackets show the detailed category in MBGD.

*^2^ Since function category was not shown in MBGD, data was obtained from the databases, COG and KEGG.

^†^ For other species, only one homolog was detected.

### Phylogeny of Trichosporonales

**Fig 2:**
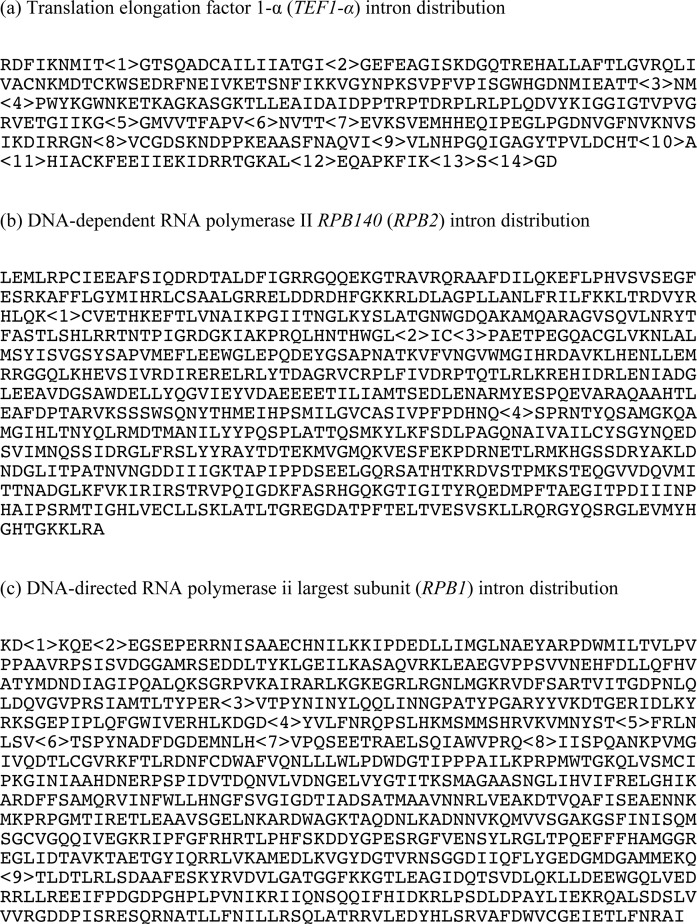
Amino acid sequences and intron positions of CDS homologs of translation elongation factor 1-α (*TEF1-α*), DNA-dependent RNA polymerase II *RPB140* (*RPB2*), and DNA-directed RNA polymerase II largest subunit (*RPB1*) from *Trichosporon asahii* JCM 2466. Numbers refer to intron positions.

First, we obtained sequence data for three regions (RNA polymerase II subunits I (*RPB1*) and II (*RPB2*) and translation elongation factor 1-α (*TEF1-α*)) that have been used for the phylogenetic analyses in Ascomycotina and Basidiomycotina [15–19]. The sequences of these regions of 12 ascomycetous yeasts (S1 Table) were obtained from the database and aligned to extract the corresponding regions from our data. The conserved domains of genes predicted by Microbial Genome Annotation Pipeline (MiGAP) (http://www.migap.org/) showed good alignment based on the codons, and we found that Trichosporonales species possessed spliceosomal introns and that their position was strain-specific. Intron positions also differed from those in *C*. *neoformans*, which was used as a model species for gene prediction using HMM-based gene finder AUGUSTUS [20] (Fig 2 and S2 Table). While the intron positions of Agaricomycetes species are sufficiently conserved to distinguish and characterize the higher taxa [16], those of Trichosporonales species are not conserved. Therefore, we identified and aligned CDS regions of *T*. *asahii* and *T*. *faecale* by referring to their mRNA data and then added genes from other species, as indicated in the Materials and Methods. The sequences used in this study covered almost the complete CDS region; 72,531 nucleotides (24,173 residues) among 30 concatenated genes. Gene alignments are available upon request.

Phylogenetic trees for the 16 species based on nucleotide and amino acid sequences using the 30 concatenated genes are shown in Fig 1b and 1c, respectively. The trees based on a single gene are presented in (S3 Table). Trees inferred from the concatenated alignments based on nucleotide (Fig 1b) and amino acid (Fig 1c) sequences had the same topology and increased reliability compared with the tree based on the D1/D2 region of the LSU rRNA gene (Fig 1a) and other single-gene trees. A phylogenetic tree inferred from the amino acid sequence alignment including consensus sequences of *T*. *coremiiforme* and *T*. *voides* (see Materials and Methods) showed an identical topology (S1 Fig). Thus, they currently represent the best estimate of the phylogenetic relationship in Trichosporonales.

To evaluate the robustness of the topology of the tree inferred from the concatenated alignments, we identified all nodes recovered during the single-gene analyses and recorded bootstrap values when the nodes were present in a tree inferred from the concatenated alignment (Table 3). Although *T*. *ovoides* should be included in the phylogenetic analyses of the *Trichosporon* genus since this is the type species of the genus, we excluded this species from the analysis, as mentioned above. Nonetheless, we continue to use the name of the clade, Ovoides, in this report.

**Table 3:** Comparison of topology between the concatenated tree and single trees.

CDS product	Homolog to: (locus_tag of *C*. *neoformans*)	Based on nucleotide sequences																
		Positions used	Highest log likelihood	Bootstrap value of each node*^1^													# nodes contained in concatenated tree*^2^	Divergence within Trichosporonales*^3^
				a	b	c	d	e	f	g	h	i	j	k	l	m		
phosphatidylinositol 3-kinase TOR1	CNF03740	7167	-71807.7691	**100**	**100**	**100**	(57)	**96**	**100**	**100**	**100**	**100**	**100**	**100**	**100**	**100**	12/13	0.2765973
glutamate synthase (NADH)	CNJ02910	6525	-58590.6785	**100**	**100**	-	**100**	**100**	**100**	**75**	**100**	**100**	**93**	**95**	**100**	**96**	12/13	0.2325986
malate synthase	CNH02910	1641	-13875.1721	**100**	**100**	**100**	-	(53)	**100**	**100**	**72**	**100**	**100**	**84**	**100**	**90**	11/13	0.2328670
phosphoenolpyruvate carboxykinase	CNI03590	1668	-10299.0077	**100**	**100**	**81**	**100**	**81**	**75**	**92**	-	**76**	**99**	-	**100**	**97**	11/13	0.1416784
ATP-binding cassette (ABC) transporter	CND00300	1932	-14912.2764	**100**	**100**	**100**	**81**	-	**100**	**91**	(38)	**100**	**100**	-	**100**	**98**	10/13	0.1990241
glycogen synthase kinase 3	CNB00720	1272	-9228.1996	**100**	**100**	**100**	-	-	**99**	**87**	-	**100**	**73**	-	**100**	**100**	9/13	0.1819497
DNA-directed RNA polymerase ii largest subunit, putative (RPB1)	CNE03720	5535	-54837.8043	**100**	**100**	**100**	-	-	**100**	**98**	-	**99**	**100**	-	**100**	**100**	9/13	0.2865913
DNA replication licensing factor cdc19 (cell division control protein 19)	CNG02380	2928	-29876.1716	**100**	**100**	**100**	**73**	-	**100**	-	**100**	-	**92**	-	**100**	**94**	9/13	0.3063356
isocitrate lyase	CNH03280	1698	-15133.6988	**100**	**100**	**100**	-	-	**87**	**80**	**71**	**98**	-	-	**100**	**100**	9/13	0.2597505
DNA-directed RNA polymerase	CNI00420	3414	-31208.3767	**87**	**100**	**100**	(48)	-	**93**	(63)	**99**	**76**	**76**	(55)	**100**	**100**	9/13	0.2359366
methylenetetrahydrofolate reductase (NADPH)	CNL04820	1929	-16548.0025	**100**	**100**	**100**	(53)	**85**	**100**	-	**99**	**99**	**99**	-	**100**	-	9/13	0.2302595
ATP dependent DNA helicase	CNB05360	2235	-22405.5341	**100**	**100**	**100**	-	(24)	**100**	**72**	**79**	**82**	(45)	(2)	**100**	-	8/13	0.2732160
adenosylhomocysteinase	CND00240	1347	-8971.2698	**98**	**100**	**100**	-	-	**82**	**90**	**76**	-	-	-	**98**	**99**	8/13	0.1546073
DNA-dependent RNA polymerase II RPB140 (RPB2)	CND03540	3849	-33545.1855	**100**	**100**	**100**	-	-	**100**	**99**	**100**	(62)	-	-	**100**	**96**	8/13	0.2237954
pyruvate carboxylase	CNF00650	3729	-28326.3212	**100**	**100**	**100**	-	(37)	**92**	(65)	**100**	**100**	-	-	**100**	**100**	8/13	0.1847746
Eukaryotic translation initiation factor 3 subunit 6	CNL05160	1920	-16255.6032	**100**	**100**	**100**	-	-	**100**	-	**100**	**100**	-	-	**100**	**99**	8/13	0.2257396
plasma membrane H(+)-ATPase 1	CNN01260	3135	-20529.9850	**100**	**100**	**99**	-	-	**100**	**93**	(62)	**100**	**77**	-	**100**	-	8/13	0.1515828
1,3-beta-glucan synthase	CNN02320	5511	-47328.6072	**100**	**100**	**100**	-	-	**100**	**100**	**100**	**100**	(46)	-	**100**	-	8/13	0.2236312
mitogen-activated protein kinase	CNC06590	1107	-8324.0754	**100**	**99**	**97**	-	-	**81**	**99**	(62)	**83**	(43)	-	**98**	(68)	7/13	0.1666310
orotidine monophosphate pyrophosphorylase (URA5)	CNG03730	687	-6763.7318	**100**	**100**	**100**	**98**	-	**91**	-	-	(53)	(63)	-	**100**	**99**	7/13	0.3037264
vacuolar ATP synthase	CNI01180	1587	-11933.2227	**100**	**100**	**89**	-	-	(52)	**75**	(37)	**99**	-	-	**100**	**97**	7/13	0.1767209
Ras2	CNJ01920	1101	-6180.5535	**99**	**100**	**99**	-	-	**97**	-	**88**	-	-	-	**100**	**99**	7/13	0.1254647
chaperone	CNM01520	2139	-13392.3250	**100**	**100**	**100**	-	(39)	**100**	-	**96**	-	-	-	**100**	**100**	7/13	0.1363423
citrate synthase	CNA00510	1416	-9234.5688	**100**	**100**	-	-	(57)	**89**	(52)	-	**93**	**98**	(69)	**100**	-	6/13	0.1478656
actin	CNA04650	1161	-5631.8845	**98**	**100**	**73**	-	-	-	**74**	(63)	**85**	-	-	**99**	(62)	6/13	0.0915010
alpha tubulin	CNB02810	1371	-7641.2544	**100**	**97**	**98**	-	(54)	**89**	-	-	-	-	(65)	**100**	**94**	6/13	0.1094242
argininosuccinate lyase	CNC04420	1416	-11523.0058	**100**	**100**	**88**	**100**	-	-	(31)	-	-	**73**	-	**100**	-	6/13	0.2090371
beta tubulin	CNC03260	1377	-7299.8992	**100**	**100**	**100**	-	-	**90**	-	-	-	-	-	**92**	-	5/13	0.1023791
adenylyl-sulfate kinase	CNE03380	641	-5714.2900	**100**	-	**96**	-	-	(57)	**92**	-	(41)	-	-	**100**	**98**	5/13	0.2429367
translation elongation factor 1-α	CNM01300	1389	-7333.6338	-	**100**	(60)	-	-	**100**	**98**	(39)	-	-	-	**96**	-	4/13	0.0985092
# nodes contained in concatenated tree*^2^				29/30	29/30	27/30	6/30	4/30	26/30	18/30	15/30	19/30	13/30	3/30	30/30	20/30		
CDS product	Homolog to: (locus_tag of *C*. *neoformans*)	Based on amino acid sequences																
		Positions used	Highest log likelihood	Bootstrap value of each node*^1^													# nodes contained in concatenated tree*^2^	Divergence within Trichosporonales*^4^
				n	o	p	q	r	s	t	u	v	w	x	y	z		
phosphatidylinositol 3-kinase TOR1	CNF03740	2388	-20501.3857	**100**	**100**	**100**	**100**	**98**	**100**	**93**	**100**	**100**	**100**	**99**	**100**	**100**	13/13	0.1414040
DNA-directed RNA polymerase ii largest subunit, putative (RPB1)	CNE03720	1844	-19055.5663	**100**	**100**	**100**	**100**	-	**100**	**80**	-	**100**	**85**	**84**	**91**	**100**	11/13	0.1861724
glutamate synthase (NADH)	CNJ02910	2174	-23206.1602	**100**	**100**	**100**	**100**	(42)	**100**	**80**	**91**	**100**	**100**	(38)	**100**	**100**	11/13	0.1956882
malate synthase	CNH02910	546	-6042.9704	**100**	**100**	**100**	-	(41)	**97**	**96**	(63)	**91**	**97**	**73**	**100**	**98**	10/13	0.2240399
methylenetetrahydrofolate reductase (NADPH)	CNL04820	633	-5915.2895	**100**	**100**	**100**	**80**	(59)	**100**	**75**	**100**	**98**	**90**	-	-	**99**	10/13	0.1540453
ATP-binding cassette (ABC) transporter	CND00300	643	-5438.2601	**97**	**100**	**100**	(40)	-	**99**	**90**	(49)	**100**	**97**	(53)	**98**	**100**	9/13	0.1424313
DNA-directed RNA polymerase	CNI00420	1137	-8524.1458	**86**	**100**	**100**	(65)	-	**96**	**78**	-	**100**	**96**	-	**99**	**100**	9/13	0.1097242
1,3-beta-glucan synthase	CNN02320	1836	-17034.1009	**97**	**100**	**100**	-	-	**100**	**99**	**100**	**100**	(24)	(51)	**100**	**100**	9/13	0.1593036
pyruvate carboxylase	CNF00650	1242	-10659.4494	**99**	**100**	**100**	-	(42)	(60)	**87**	**100**	**100**	-	-	**100**	**100**	8/13	0.1286247
DNA replication licensing factor cdc19 (cell division control protein 19)	CNG02380	975	-11547.8857	**97**	**100**	**100**	(60)	-	**96**	-	**100**	**71**	(43)	-	**96**	**100**	8/13	0.2288008
isocitrate lyase	CNH03280	565	-6484.3719	**99**	**100**	**100**	(64)	-	**94**	-	**82**	**93**	-	-	**98**	**100**	8/13	0.2222622
vacuolar ATP synthase	CNI01180	528	-3637.3220	**96**	**99**	-	(63)	-	**95**	**94**	(53)	**79**	**70**	-	**85**	**100**	8/13	0.0735907
phosphoenolpyruvate carboxykinase	CNI03590	555	-3829.0758	**97**	**100**	**92**	**85**	-	(44)	-	-	**89**	**97**	(56)	**97**	**100**	8/13	0.0931894
plasma membrane H(+)-ATPase 1	CNN01260	1044	-8907.8908	**100**	**100**	**100**	-	(47)	**100**	**77**	(54)	**100**	(62)	-	**98**	**100**	8/13	0.1269335
ATP dependent DNA helicase	CNB05360	744	-7343.5154	**100**	**100**	**100**	(36)	-	**99**	-	(29)	**95**	(50)	-	**74**	**100**	7/13	0.1617158
adenosylhomocysteinase	CND00240	447	-4136.0745	(24)	**100**	**91**	-	-	**72**	**93**	**71**	-	-	-	**77**	**100**	7/13	0.1579080
DNA-dependent RNA polymerase II RPB140 (RPB2)	CND03540	1280	-10617.4474	**99**	**100**	**99**	-	-	**100**	-	(63)	**100**	-	-	**96**	**100**	7/13	0.1122744
orotidine monophosphate pyrophosphorylase (URA5)	CNG03730	228	-2840.2696	**100**	**99**	**97**	**96**	-	**86**	-	-	(69)	(33)	-	**100**	**100**	7/13	0.2999401
Ras2	CNJ01920	366	-2531.3792	**97**	**100**	**94**	(55)	-	**95**	-	**90**	-	-	-	**100**	**100**	7/13	0.0971751
Eukaryotic translation initiation factor 3 subunit 6	CNL05160	639	-6146.9708	**97**	**100**	**95**	-	-	**100**	(61)	**76**	**100**	-	-	-	**100**	7/13	0.1704422
chaperone	CNM01520	712	-5502.4940	**100**	**100**	**99**	-	-	**92**	(59)	**86**	**70**	-	-	(61)	**100**	7/13	0.1150690
glycogen synthase kinase 3	CNB00720	421	-2686.2647	**88**	**76**	**95**	-	-	(52)	-	(62)	**100**	-	-	**98**	**100**	6/13	0.0792372
argininosuccinate lyase	CNC04420	471	-4055.3207	**91**	**100**	**95**	**99**	-	**96**	-	(58)	-	-	(59)	-	**100**	6/13	0.1375764
actin	CNA04650	386	-1520.7293	**82**	**93**	**84**	-	-	-	-	(69)	**96**	-	-	-	**99**	5/13	0.0254971
beta tubulin	CNC03260	458	-2074.0608	**93**	**100**	**98**	-	-	(61)	-	**90**	-	-	-	(50)	**100**	5/13	0.0307975
mitogen-activated protein kinase	CNC06590	368	-1562.5243	**97**	**81**	**100**	(68)	-	**89**	-	-	(63)	(42)	-	(54)	**74**	5/13	0.0313906
adenylyl-sulfate kinase	CNE03380	212	-2039.7504	**98**	**80**	**77**	-	-	(62)	-	**74**	(49)	-	-	(59)	**97**	5/13	0.1536762
citrate synthase	CNA00510	471	-4170.0870	**100**	**100**	(67)	-	-	(31)	-	(19)	(49)	(57)	-	**100**	**71**	4/13	0.1482440
translation elongation factor 1-α	CNM01300	462	-2674.2830	(56)	**100**	-	-	-	**94**	**99**	(44)	-	-	-	-	**97**	4/13	0.0626686
alpha tubulin	CNB02810	456	-2114.6945	**100**	-	-	-	(48)	**83**	-	-	-	-	(21)	(39)	**89**	3/13	0.0321153
# nodes contained in concatenated tree*^2^				28/30	29/30	26/30	7/30	1/30	23/30	13/30	13/30	20/30	9/30	3/30	20/30	30/30		

*^1^, Node names are shown in Fig 1b and 1c. Numerals show the bootstrap value of each node which corresponded to that of the concatenated tree [boldface, more than 70%; (), less than 70%].—indicates that the node on the concatenated tree is not shown in respective CDS tree.

*^2^, Nodes showing more than 70% bootstrap value (boldface) were treated as "positive".

*^3^, The number of base substitutions per site from averaging over all sequence pairs are shown. Analyses were conducted using the Kimura 2-parameter model [47]. All ambiguous positions were removed for each sequence pair.

*^4^, The number of amino acid substitutions per site from averaging over all sequence pairs are shown. Analyses were conducted using the JTT matrix-based model [45]. All ambiguous positions were removed for each sequence pair.

In the Ovoides clade, *T*. *asahii*, *T*. *faecale* and *T*. *inkin* formed a subclade, as shown by the high bootstrap values in the single-gene trees for the majority of genes [nodes a (29/30) and b (29/30) in Fig 1b and n (28/30) and o (29/30) in Fig 1c and Table 3, and hereafter]. Regarding the Cutaneum clade, the two species examined (*T*. *cutaneum* and *T*. *dermatis*) formed a cluster in trees inferred from the concatenated alignment, which was also observed in single-gene trees [nodes c (27/30) and p (26/30)]. The Ovoides and Cutaneum clades formed a monophyletic group, which was supported with 100% and 72% bootstrap values for nucleotide and amino acid sequences, respectively. The tree based on the D1/D2 region of the LSU rRNA gene did not support this cluster (Fig 1a), and single-gene trees showing more than 70% bootstrap values were 4 of 30 [nodes e] and 1 of 30 [node r] based on nucleotide and amino acid sequences, respectively.

The Porosum and Gracile clades formed a cluster, and the connection of the Brassicae clade was shown in the tree based on the D1/D2 region of the LSU rRNA gene (Fig 1a). In trees inferred from the concatenated alignment, the Brassicae and Gracile clades were clustered and connected to the Porosum clade with 100% bootstrap values for both nucleotide and amino acid sequences. The bootstrap values for single-gene trees were similar to those for trees inferred from the concatenated alignment: two-thirds supported the connection of the Brassicae and Gracile clades [nodes i (19/30) and v (20/30)], and one-third supported their connection to the Porosum clade [nodes j (13/30) and w (9/30)].

*Cryptococcus curvatus* is in an adjunct position in the Cutaneum clade with 100% bootstrap values for both nucleotide and amino acid sequences (Fig 1b and 1c). We previously evaluated the phylogenetic position of the species based on rRNA genes and spacer regions, but were unable to obtain good resolution [21–22]. In this study, the single-gene trees did not frequently support the node [more than 70% bootstrap support nodes for d (6/30) and q (7/30)].

*Vanrija humicola* was located outside of the *Trichosporon* species, which has been reported previously [2, 5, 6, 21, 23]. As described above, Weiss et al. [6] proposed the use of genus *Vanrijia* as the genus name, and we follow their suggestion in this report. The genus *Vanrija sensu* Moore [7] is polyphyletic; thus the emendation and subsequent reclassification including new combinations is required. We believe that our genes will contribute to the reclassification; namely, delineation for the species at the basal position between *Vanrija* and other genera.

The *Bullera koratensis* and *Cryptococcus tepidarius* clades occurred at remote positions in the Trichosporonales. In addition, the intron positions were closer to those of *C*. *neoformans* than to those of other *Trichosporon* species (S2 Table). Okoli et al. [3] proposed the genus *Cryptotrichosporon* based on the D1/D2 region of the LSU rRNA plus ITS regions and the 18S rRNA gene, and noted that *B*. *koratensis* and *C*. *tepidarius* were phylogenetically closely related to their species. These species might be pivotal in clarifying the phylogenetic relationships between Tremellales and Trichosporonales, since they obtain intermediate positions.

### Phenotypic properties and phylogeny

*Trichosporon* has been divided into five clades based on a combination of serological properties and major ubiquinone as described above [2, 24]. Based on morphological aspects, species in the clades Brassicae, Cutaneum, Gracile, and Ovoides produce abundant arthroconidia, whereas those in the Porosum clade rarely produce arthroconidia or not at all [2]. Fig 1b and 1c showed clustering of the Brassicae and Gracile clades and a connection to the Porosum clade with 100% bootstrap values for both nucleotide and amino acid sequences, indicating that this morphological characteristic supports the linkage between Brassicae and Gracile clades. Recently, Nagy et al. [25] reported that Zn-finger transcription factor families are related to switches between yeast and filamentous forms. Since the production of arthroconidium has been reported in the genera *Guehomyces* and *Tausonia* (Cystofilobasidiales) and species in the family Dipodascaceae (Saccharomycetales), genes associated with arthroconidium production may be found when the genomic data are accumulated.

Regarding serological properties, five clades; namely, Cutaneum, Ovoides, Brassicae, Gracile and Porosum, are characterized by serogroup I, II, III, I-III and I-III (reacting with both factors I and III), respectively [26]. The clustering topology of clades Brassicae (serogroup I) and Gracile (I-III), and the connection to clade Porosum (I-III), indicated that these three clades can be characterized by positive reactions with III as common antisera (S2 Fig). This grouping, serogroups I, II and III, was proposed previously [27] and confirmed in this study. The epitopes of serological group II and III have been reported [28–29]; thus, this characteristic would be a useful property for differentiation.

For the major ubiquinone in ascomycetous yeasts, ubiquinone isoprene chain length ranged from 6 to 10 (namely Q-6 to Q-10) and showed a strong relationship with phylogeny of higher taxa with some exceptions, and was used as a phenotypic characteristic [15, 30]. In basidiomycetous yeasts, most species contain Q-10 and few genera are characterized by different chain lengths: *Mrakia* and *Cystofilobasidium* (both in Cystofilobasidiales) are characterized by Q-8, and Q-9 was observed in *Trichosporon* (excluding the Cutaneum clade), *Vanrija*, *Guehomyces*, and *Itersonilia* in Agaricomycotina. In this study, Q-10 species *C*. *curvatus* clustered with the Cutaneum clade, which has been characterized by Q-10 (S2 Fig), and this characteristic supports this linkage. Although the taxonomic significance of ubiquinone isoprene chain length remains unclear due to the occurrence of both Q-9 and Q-10 in the same genus; e.g., in *Leucosporidium* [31], this could be used as a distinctive characteristic with serological properties when the Cutaneum clade together with *C*. *curvatus* is proposed as a separate genus. We believe that further studies will clarify how the isoprene chain length is controlled by prenyltransferase and related mechanisms.

### Clarification of the topology of basal species in a lineage by obtaining a highly resolved tree

Recently, the number of fungal phylogenetic trees based on genomic data have increased due to the greater amount of genomic data available e.g., [32–36]. However, studies on fungi are still fewer compared with bacteria and archaea, mainly because of the amount of available genomic data. Based on the NCBI BioProject (http://www.ncbi.nlm.nih.gov/bioproject/), only 84 and 230 species were available for yeasts and filamentous fungi, respectively, as of May 15th, 2014. With the available fungal genomic data, Rokas et al. [32] demonstrated that a minimum of 20 concatenated genes in an analysis were sufficient to represent the phylogeny based on all genomic data for *Saccharomyces* species. Kuramae et al. [33] showed that 40–45 concatenated proteins would be required to resolve the tree of life of the fungal kingdom by cophenetic correlation analysis. Based on these reports, we believe that concatenated analyses of genes could be used for robust studies on the fungal tree of life, although the number of genes required would depend on the range of the target groups.

Rokas et al. [32] used 106 genes and compared a total length of 127,026 nucleotides (42,342 residues), which corresponded to roughly 1% of the genomic sequences and 2% of the predicted genes. According to this estimation, the use of “a concatenated analysis of 20 or more genes”, corresponding to roughly 0.2% of the genomic sequences, is sufficient to construct an accurate phylogeny. Our data based on 72,531 nucleotides and corresponding to 0.24% (for *Trichosporon laibachii*, genome size 30.6 Mb) to 0.40% (for *C*. *curvatus*, genome size 18.2 Mb) of the total genomic sequences of Trichosporonales support the results of Rokas et al. [32].

While the number of genes required to represent a tree with a resolution comparable to the resolution of a genome tree has been discussed [32, 34], an example of such a set of genes has not been reported. In this study, we propose a set of genes suitable for constructing a phylogenetic tree with high resolution to examine the evolutionary diversification in Trichosporonales. Table 3 indicates that the use of more genes does not improve the resolution of the tree of this taxonomic group. In addition, among the nucleotide sequences used in this study, those for four genes (phosphatidylinositol 3-kinase *TOR1*, glutamate synthase (NADH), malate synthase and phosphoenolpyruvate carboxykinase) showed better resolution than those typically used for multigene analyses, such as *TEF1-α*, *RPB1* and *RPB2*. For the tree based on amino acid sequences of phosphatidylinositol-3-kinase *TOR1* (even if used alone), the same topology was obtained as for the tree inferred from the concatenated alignment. The tree inferred from the concatenated alignment based on *RPB1*, *RPB2* and *TEF1-α* (regions corresponding to those used by Kurtzman and Robnett [15]) revealed the existence of five clades in *Trichosporon* (nodes b, c, f, g and h) with 100% bootstrap values and a clear relationship among the Brassicae, Gracile and Porosum clades (nodes i and j; S3 Fig). This tree had better resolution than the respective single-gene trees, as shown in (S4 Table), but did not clarify the relationship between the Ovoides clade and the other four *Trichosporon* clades (nodes e and r), or the positions of *V*. *humicola* (nodes k and x) and *C*. *curvatus* (node d). Obviously, the use of these three genes is not enough to clarify the phylogenetic relationship of the Trichosporonales. This was our original motivation for the present study.

In yeasts, resolution of the phylogenetic relationships has particularly been increased for the ascomycetous teleomorphic genera using partial sequences of *TEF1-α* and cytochrome oxidase II, in addition to rRNA genes. The later addition of *RPB1* and *RPB2* improved the resolution for the Saccharomycetales yeast species [15]. Recently, Koufopanou et al. [37] reported primer sets for 14 genes to clarify the topology of basal species among the Saccharomycetales. Of our 30 genes, glutamate synthase and adenosyl homocysteinase were used for multiple gene analysis by Koufopanou et al. [37], and *CDC19* was selected as a candidate during the first screening stage but was not used in the published analysis. Gene duplication in the Tor pathway was also reported in some fungi by Shertz et al. [38]. Careful attention must be paid when conducting multigene analyses in other taxa using the Tor gene; however, this gene can be used for Trichosporonales, since we did not detect a Tor paralog in our experiments.

## Conclusions

In this study, phylogenetic trees inferred from concatenated alignments (nucleotide and amino acid sequences) of 30 genes extracted from draft genome data showed greater resolution than in previous reports, indicating that they currently represent the best estimate of the phylogenetic relationship in Trichosporonales. In addition, we suggest that the genes identified in this study are good candidates for constructing a multi-locus sequence analysis system for both inter- and intra-species analyses. Specific primers are required to adopt these newly discovered promising genes. These genes can also be used for epidemiological and biogeographical studies. A set of genes suitable for constructing a phylogenetic tree to examine evolutionary diversification. The relationship between the closely related Trichosporonales and Tremellales in the Agaricomycotina might be clarified using these genes [6, 17, 39]. Moreover, our study provides a platform for future studies on a comprehensive reclassification of pleomorphic fungi.

## Materials and Methods

### Genome sequencing

#### Strains and DNA and RNA extraction

Strains used in this study were received from the Japan Collection of Microorganisms at the RIKEN Bioresource Center (Table 1). Cultures grown on YM agar (Difco, Detroit, MI; BD, Franklin Lakes, NJ, USA) were collected and freeze-dried, and DNA was prepared according to a previous method [40]. The integrity and quality of the extracted genomic DNA were examined by agarose gel electrophoresis using a NanoDrop 2000 spectrophotometer (Thermo Scientific, Wilmington, DE, USA). The concentration of DNA was determined using a Quant-iT PicoGreen dsDNA Assay Kit (Life Technologies, Gaithersburg, MD, USA). Total RNA was prepared from *T*. *asahii* and *T*. *faecale* cells cultivated at 37°C or 28°C using an RNeasy Mini Kit (Qiagen, Hilden, Germany) according to the manufacturer’s instructions after disruption by glass beads. The integrity of the RNA was verified by agarose gel electrophoresis. The RNA concentration was determined using a NanoDrop 2000 spectrophotometer.

#### Genome sequencing

Draft genomes of 17 selected species in the Trichosporonales were determined using a 454 GS FLX (Roche Diagnostics, Mannheim, Germany) and/or Illumina (Illumina Inc., San Diego, CA, USA) next-generation sequencer. Since karyotyping data is not available for the Trichosporonales, we were unable to estimate the genome sizes of these species before the beginning of this study. Thus, the genome size of *C*. *neoformans* (approximately 20 Mb) was used as an initial estimate.

DNA library preparation and sequencing were performed according to the manufacturer’s instructions. Briefly, a paired-end DNA library was prepared from 1 μg genomic DNA, following DNA fragmentation into approximately 350-bp average sized fragments using an S2 ultrasonicator (Covaris, Woburn, MA, USA) and then sequenced using a HiSeq 2000 sequencer (Illumina Inc.; S5 Table). The *T*. *asahii* genome was also sequenced using the GS FLX Titanium platform (Roche Diagnostics). A DNA library was prepared from 0.6 μg *T*. *asahii* genomic DNA, followed by fragmentation into approximately 900-bp average sized fragments using a Covaris S2 ultrasonicator and then sequencing using GS FLX pyrosequencing technologies. *De novo* assembly was performed using the CLC Genomics Workbench (version 4.5 or later) and CLC Genomics Server (version 3.6 or later) (CLC Bio Co., Aarhus, Denmark) with the default settings, including the “Update contigs” option, which edited contig sequences based on the reads mapped to the contig. The *de novo* assembly produced contigs with an average N50 value of 37,614. Key attributes of the draft genome sequences are summarized in Table 1. Library generation, sequencing and *de novo* assembly were performed at the Genome Network Analysis Support Facility, RIKEN CLST (Yokohama, Japan).

#### Transcriptomic sequencing

An RNA-Seq library was prepared as described previously [41]. Briefly, polyA(+) RNA was extracted from 10 μg of total RNA using Dynabeads Oligo(dT)25 (Life Technologies), according to the manufacturer’s protocols, and was fragmented by heating at 70°C for 3.5 min in 0.5× fragmentation buffer (Life Technologies). Fragmented RNA was purified using the RNeasy MinElute Kit (Qiagen) and then dephosphorylated using Antarctic phosphatase (New England Biolabs, Beverly, MA, USA). The dephosphorylated RNA was phosphorylated using T4 polynucleotide kinase (New England Biolabs) with 10 mM ATP and was purified again using the RNeasy MinElute Kit. Purified RNA was concentrated and ligated at both ends with 3’ and 5’ adapters from the TruSeq Small RNA Sample Prep Kit (Illumina Inc.) using T4 RNA ligase 2 and T4 RNA ligase 1 (New England Biolabs), respectively. Adapted RNA was reverse transcribed using PrimeScript Reverse Transcriptase (Takara Bio, Kyoto, Japan) and amplified using Phusion High-Fidelity DNA Polymerase (New England Biolabs), as well as forward and reverse primers from the TruSeq Small RNA Sample Prep Kit. cDNA libraries with a size range of 160–320 bp were selected by removing PCR primers using 1.2 volumes of AMPure XP beads (Beckman Coulter, Miami, FL, USA) and subsequent gel extraction using Pippin Prep (Sage Science, Beverly, MA, USA). The four resulting cDNA libraries were pooled and then subjected to 101-bp single-read sequencing on a HiSeq 2000 sequencer. The sequencing produced 97 million and 88 million reads for *T*. *asahii* cultivated at 37°C and 28°C, respectively, and 80 million and 117 million reads for *T*. *faecale* cultivated at 37°C and 28°C, respectively. The reads were trimmed of adapter, low-quality and ambiguous sequences using the CLC Genomics Workbench with default parameters and were then assembled *de novo* with the CLC Genomics Workbench and CLC Genomics Server. The draft transcriptome assemblies of *T*. *asahii* at 37°C and 28°C and of *T*. *faecale* at 37°C and 28°C consisted of 26,569 (N50 = 1,307), 26,769 (N50 = 1,382), 24,105 (N50 = 1,362) and 25,349 (N50 = 1,389) transcripts, respectively. Library generation, sequencing, and *de novo* assembly were performed at the Genome Network Analysis Support Facility, RIKEN CLST.

#### Auto-annotation

After the *de novo* assembly, auto-annotation was performed using MiGAP (http://www.migap.org/), a publicly available annotation pipeline system of microbial and fungal genome draft sequences operated by the National Institute of Genetics. Briefly, MiGAP provides consecutive gene searching and annotation functions and consists of prediction programs. Exons and introns were predicted using the HMM software AUGUSTUS 2.5.5 [20]. The HMM model species used for this prediction is cryptococcus_neoformans_neoformans_JEC21. After gene prediction, each ORF was subjected to a homology search against the following protein sequence databases using NCBI BLAST: the fungi division of RefSeq, whole entries of TrEMBL and whole entries of NR.

### Alignment and phylogenetic analyses

#### Gene selection and alignment

For selection of genes, we used genomic data from *C*. *neoformans* (AE017341) and *C*. *gattii* (CP000286) as references. Since our target genes were those that can be used for genus-level discrimination, they were required to be conserved in the same genus. A total of 1,234 CDSs were selected from *C*. *neoformans*, *C*. *gattii* and *T*. *asahii* genomic data under the conditions of identity = 60 and coverage = 60, using in-silico molecular cloning (IMC; In Silico Biology, Inc., Yokohama, Japan). CDS homologs showing more than 97% similarity between *C*. *neoformans* and *C*. *gattii* were listed. Candidate *T*. *asahii* genes were selected using IMC under the criteria that they include approximately 300 or more amino acids and do not include many introns, since our purpose was to develop signature/marker sequences. CDS homologs of other species were then selected using IMC. Genes used in this study are listed in Table 2 with DDBJ/GenBank/EMBL accession numbers.

The coding regions of genes from *T*. *asahii* and *T*. *faecale* were determined using a BLAST search against the respective mRNA data. Multiple alignment of respective genes was first performed using the CDSs of *T*. *asahii* and *T*. *faecale* using those of *C*. *neoformans* as an outgroup. Subsequently those of other species were added and aligned based on codons. In some cases, part of a gene could be found in one contig and the other parts in different contigs. In this case, manual alignment was performed.

When making an alignment file, the following treatment was performed for two CDSs beforehand: An obvious insertion (1,557 nucleotides) in CND03540 (DNA-dependent RNA polymerase II (*RPB2*)) homolog was found in *T*. *asahii* but not in other species, and the region was eliminated from the analyses. Since the same was observed in the published *T*. *asahii* genome data (for both ALBS01000000 and AMBO01000000), this insertion was assumed to be distinct to this species or these strains in Trichosporonales. For CNF03740 (phosphatidylinositol-3-kinase *TOR1*) homolog, the length of CDSs of 9 species studied, viz. *Trichosporon brassicae*, *T*. *cutaneum*, *T*. *dermatis*, *Trichosporon scarabaeorum*, *B*. *koratensis*, *C*. *curvatus*, *C*. *tepidarius*, *V*. *humicola* and *C*. *neofomans* (outgroup) were 7,167 nucleotides (2,388 amino acids), whereas those of other species were found to be longer and varied. Since they aligned well from the 5’ end to position 7,167 by codon, 7,167 nucleotides (2,388 amino acids) were used for the analyses; the downstream sequences were not used. Since CDSs were orthologous genes shown in the Microbial Genome Database for Comparative Analysis (MBGD) (http://mbgd.nibb.ac.jp), CDSs were concatenated using SeaView [42].

#### Alignment including the consensus amino acid sequence of *T*. *coremiiforme* and *T*. *ovoides*

As shown in Table 2, we found paralogous genes in *T*. *ovoides* and *T*. *coremiiforme*. In this case, we created a consensus sequence from paralogs (from a multiple alignment) with X indicating a non-identical amino acid. When a partial sequence of gene was found only in one of the two sequences, this partial sequence was included in the consensus sequence.

#### Alignment of frequently used regions of *RPB1*, *RPB2* and *TEF1-α* in fungi

Sequence alignments of *RPB1*, *RPB2*, and *TEF1-α* that are often used for the phylogenetic analyses of fungi were generated by referring to sequences of 12 ascomycetous yeasts [15]. The reference sequences used for this extraction are shown in (S1 Table).

#### Alignment of the D1/D2 region of LSU rRNA gene

All sequences of the D1/D2 region of LSU rRNA gene were obtained from GenBank/EMBL/DDBJ database (S1 Table). A multiple alignment was performed using MEGA6 [43] and edited manually.

#### Phylogenetic analyses

Trees (a phylogenetic tree based on each gene and a tree inferred from the concatenated alignment) were constructed using the maximum likelihood method in MEGA6 [43]. When a tree was constructed based on nucleotide sequences, the Tamura-Nei model [44] was used for the analyses with options Rates and Patterns = uniform rates. Initial trees for the heuristic search were obtained automatically by applying the neighbor-joining and BIONJ algorithms to a matrix of pairwise distances estimated using the maximum composite likelihood (MCL) approach and then selecting the topology with the highest log likelihood value. When a tree was inferred based on amino acid sequences, the JTT matrix-based model [45] was used for analyses with option Rates and Patters = uniform dates. Initial trees for the heuristic search were obtained automatically by applying the neighbor-joining and BIONJ algorithms to a matrix of pairwise distances estimated using a JTT model and then selecting the topology with the highest log likelihood value. All sites were used in all analysis with 100 times bootstrap analyses [46].

Average base substitutions per site and average amino acid substitutions per site for each gene were conducted using the Kimura 2-parameter model [47] and the JTT matrix-based model [45], respectively.

## Supporting Information

S1 Table Sequence accession numbers retrieved from the GenBank/EMBL/DDBJ databases in this study. (XLSX)

S2 Table Intron distribution in translation elongation factor 1-α (*TEF1-α*), DNA-directed RNA polymerase ii largest subunit (*RPB1*) and DNA-dependent RNA polymerase II (*RPB2*). (XLSX)

S3 Table Phylogenetic trees based on amino acid and nucleotide sequences. (XLSX)

S4 Table Comparison of topology between the 30 gene concatenated tree and single and concatenated tree of the *RPB1*, *RPB2* and *TEF1-α* genes. (XLSX)

S5 Table Basic statistics of whole-genome shotgun sequencing. (XLSX)

S1 Fig Phylogenetic tree inferred from the amino acids sequences including consensus sequence of *T*. *coremiiforme* and *T*. *ovoides*. The amino acid consensus sequences for *T*. *coremiiforme* and *T*. *ovoides* were created (see Materials and Methods) and labeled “consensus” in parenthesis following the species name. The tree was constructed using the maximum likelihood method based on a JTT matrix-based model [45]. Numerals on each node represent the percentages from 100 replicating bootstrap samplings [46]. There were a total of 24,173 amino acids in the dataset, and the tree with the highest log likelihood (-219231.8349) is shown. *B*., *Bullera*; *C*., *Cryptococcus*; *T*., *Trichosporon*; *V*., *Vanrija*.
(TIF)

S2 Fig Phylogenetic tree of Trichosporonales species with phenotypic properties. Phylogenetic tree in Fig 1b is shown with phenotypic properties [2]. *B*., *Bullera*; *C*., *Cryptococcus*; *T*., *Trichosporon*; *V*., *Vanrija*.
(TIF)

S3 Fig Phylogenetic tree of Trichosporonales species based on concatenated partial sequences of *RPB1*, *RPB2* and *TEF1-α* genes. (a) Nucleotide sequences and (b) amino acid sequences. The trees in (a) and (b) were constructed using the maximum likelihood method based on the Tamura-Nei model [44] for (a) and a JTT matrix-based model [45] for (b). Numerals on each node represent the percentages from 100 replicating bootstrap samplings (frequencies of less than 60% are not shown) [46]. Letters in brackets after bootstrap values indicate the node name (see Table 3). Positions used: (a) 5,934 nucleotides, (b) 1,975 amino acids. Highest log likelihood: (a) -51394.7872, (b) -16428.1661. *B*., *Bullera*; *C*., *Cryptococcus*; *T*., *Trichosporon*; *V*., *Vanrija*.
(TIF)
