# Correction: Selection of Orthologous Genes for Construction of a Highly Resolved Phylogenetic Tree and Clarification of the Phylogeny of Trichosporonales Species

**DOI:** 10.1371/journal.pone.0138637

**Published:** 2015-09-15

**Authors:** The PLOS ONE Staff

The following information is missing from the Funding section: MT is also supported by the Grants-in-Aid for Scientific Research (Number 26650148) from the Japan Society for the Promotion of Science (JSPS).

The publisher apologizes for the error.
