# Supplementary figures and images for: Selection of Orthologous Genes for Construction of a Highly Resolved Phylogenetic Tree and Clarification of the Phylogeny of Trichosporonales Species
Source: PLoS One. 2015 Aug 4;10(8):e0131217. doi: 10.1371/journal.pone.0131217 (PMC4524599; doi:10.1371/journal.pone.0131217)

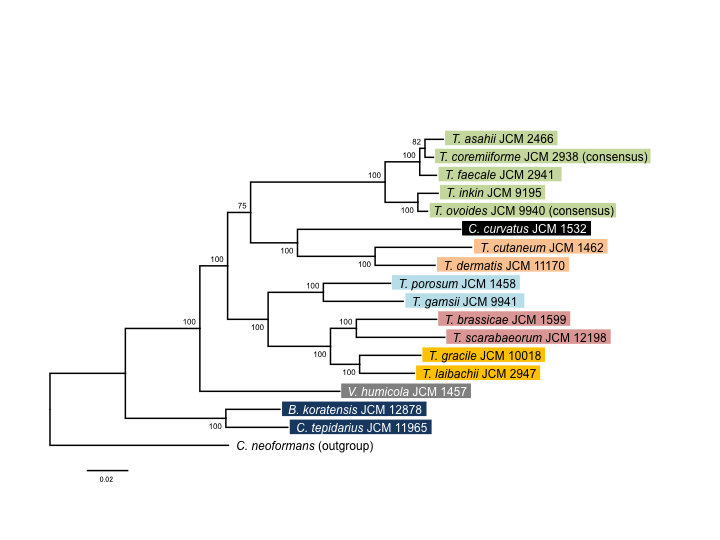

Supplement: S1 Fig — The amino acid consensus sequences for T. coremiiforme and T. ovoides were created (see Materials and Methods) and labeled “consensus” in parenthesis following the species name. The tree was constructed using the maximum likelihood method based on a JTT matrix-based model [45]. Numerals on each node represent the percentages from 100 replicating bootstrap samplings [46]. There were a total of 24,173 amino acids in the dataset, and the tree with the highest log likelihood (-219231.8349) is shown. B., Bullera; C., Cryptococcus; T., Trichosporon; V., Vanrija. (TIF) [file pone.0131217.s006.tif]

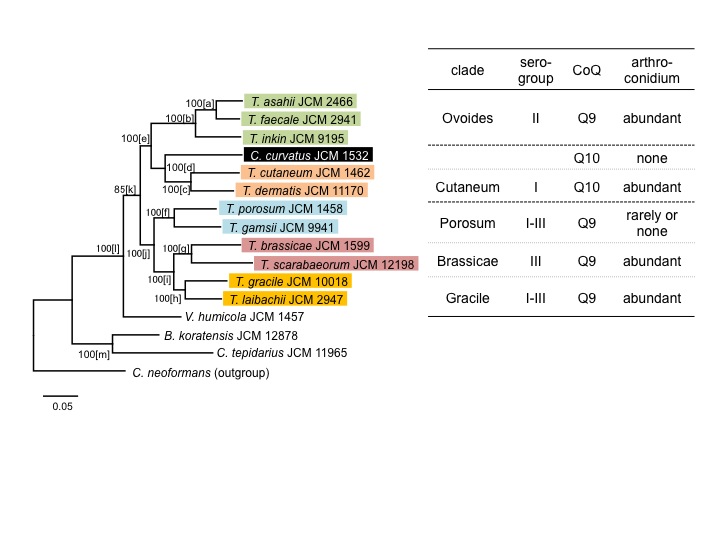

Supplement: S2 Fig — Phylogenetic tree in Fig 1b is shown with phenotypic properties [2]. B., Bullera; C., Cryptococcus; T., Trichosporon; V., Vanrija. (TIF) [file pone.0131217.s007.tif]

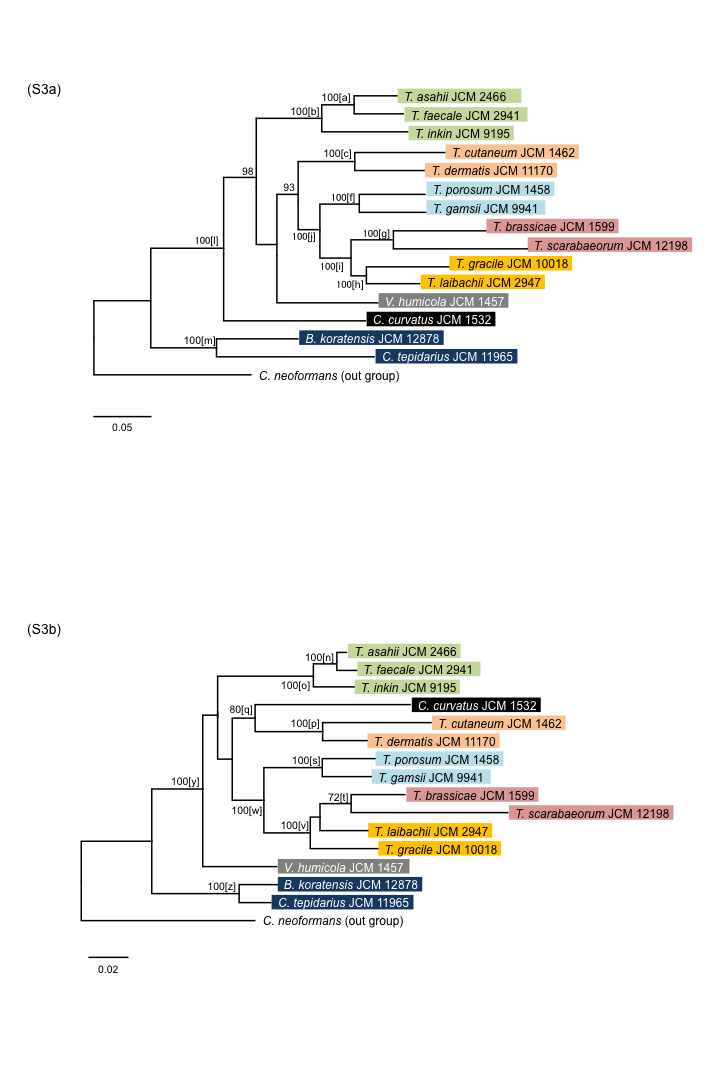

Supplement: S3 Fig — (a) Nucleotide sequences and (b) amino acid sequences. The trees in (a) and (b) were constructed using the maximum likelihood method based on the Tamura-Nei model [44] for (a) and a JTT matrix-based model [45] for (b). Numerals on each node represent the percentages from 100 replicating bootstrap samplings (frequencies of less than 60% are not shown) [46]. Letters in brackets after bootstrap values indicate the node name (see Table 3). Positions used: (a) 5,934 nucleotides, (b) 1,975 amino acids. Highest log likelihood: (a) -51394.7872, (b) -16428.1661. B., Bullera; C., Cryptococcus; T., Trichosporon; V., Vanrija. (TIF) [file pone.0131217.s008.tif]
